# Overexpression of P2X3 and P2X7 Receptors and TRPV1 Channels in Adrenomedullary Chromaffin Cells in a Rat Model of Neuropathic Pain

**DOI:** 10.3390/ijms20010155

**Published:** 2019-01-03

**Authors:** Marina Arribas-Blázquez, Luis Alcides Olivos-Oré, María Victoria Barahona, Mercedes Sánchez de la Muela, Virginia Solar, Esperanza Jiménez, Javier Gualix, J. Michael McIntosh, Antonio Ferrer-Montiel, María Teresa Miras-Portugal, Antonio R. Artalejo

**Affiliations:** 1 Department of Pharmacology and Toxicology, Veterinary Faculty, Universidad Complutense de Madrid, 28040 Madrid, Spain; marina.arribas@vet.ucm.es (M.A.-B.); olivos@ucm.es (L.A.O.-O.); vbg@ucm.es (M.V.B.); virvir93@gmail.com (V.S.); esperanzajim@gmail.com (E.J.); 2 Instituto Universitario de Investigación en Neuroquímica, Universidad Complutense de Madrid, 28040 Madrid, Spain; jgualix@ucm.es (J.G.); mtmiras@vet.ucm.es (M.T.M.-P.); 3 Department of Animal Medicine and Surgery, Veterinary Faculty, Universidad Complutense de Madrid, 28040 Madrid, Spain; sdlmuela@vet.ucm.es; 4 Department of Biochemistry and Molecular Biology, Veterinary Faculty, Universidad Complutense de Madrid, 28040 Madrid, Spain; 5 George E. Wahlen Veterans Affairs Medical Center, Salt Lake City, UT 84148, USA; mcintosh.mike@gmail.com; 6 Departments of Biology and Psychiatry, University of Utah, Salt Lake City, UT 84112, USA; 7 Instituto de Biología Molecular y Celular (IBMC), Universitas Miguel Hernández, 03202 Elche, Spain; aferrer@umh.es

**Keywords:** P2X3 receptors, P2X7 receptors, TRPV1 channels, α9 nicotinic acetylcholine receptors, neuropathic pain, chromaffin cells, adrenal medulla, stress

## Abstract

We have tested the hypothesis that neuropathic pain acting as a stressor drives functional plasticity in the sympathoadrenal system. The relation between neuropathic pain and adrenal medulla function was studied with behavioral, immunohistochemical and electrophysiological techniques in rats subjected to chronic constriction injury of the sciatic nerve. In slices of the adrenal gland from neuropathic animals, we have evidenced increased cholinergic innervation and spontaneous synaptic activity at the splanchnic nerve–chromaffin cell junction. Likewise, adrenomedullary chromaffin cells displayed enlarged acetylcholine-evoked currents with greater sensitivity to α-conotoxin RgIA, a selective blocker of α9 subunit-containing nicotinic acetylcholine receptors, as well as increased exocytosis triggered by voltage-activated Ca^2+^ entry. Altogether, these adaptations are expected to facilitate catecholamine output into the bloodstream. Last, but most intriguing, functional and immunohistochemical data indicate that P2X3 and P2X7 purinergic receptors and transient receptor potential vanilloid-1 (TRPV1) channels are overexpressed in chromaffin cells from neuropathic animals. These latter observations are reminiscent of molecular changes characteristic of peripheral sensitization of nociceptors following the lesion of a peripheral nerve, and suggest that similar phenomena can occur in other tissues, potentially contributing to behavioral manifestations of neuropathic pain.

## 1. Introduction

Neuropathic pain is a relevant clinical problem due to its severity, form of presentation (spontaneous pain, hyperalgesia and allodynia), and frequent chronic evolution. Moreover, the moderate effectiveness of current analgesics makes it an unmet medical need. Despite recent advances in the knowledge of nociceptive information processing in neuropathic pain, it is still necessary to go further into the study of the pathophysiology of this condition to facilitate the development of new more effective drugs or, at least, to repurpose some that are currently in clinical use. In this context, it is noteworthy that ATP has been involved in several forms of chronic pain acting mostly through purinergic ionotropic, P2X, receptors located both on peripheral nociceptors and second-order nociceptive neurons in the spinal cord [1]. Interestingly, the critical role that ATP seems to play in pathological pain correlates with increased activity and/or expression of ATP receptors [1], thereby contributing to peripheral and central sensitization of pain pathways. Therefore, selective targeting of specific types of P2X receptors, like P2X3, P2X4 and P2X7, constitutes a promising approach in the search for new analgesic drugs [2,3,4,5].

A related issue in the study of neuropathic pain secondary to the injury of a peripheral nerve is the contribution of the sympathetic nervous system (SNS) to pain generation and maintenance. Such a contribution is accepted when chemical (e.g., guanethidine or 6-OH-dopamine treatment) or surgical sympathectomy ameliorates clinical symptoms [6]. SNS involvement would depend on the algesic effect of noradrenaline released from postganglionic sympathetic nerve endings neighboring terminals and somata of primary nociceptive neurons (dorsal root ganglia, DRG, neurons). Interestingly, circulating catecholamines, adrenaline and noradrenaline, derived from the adrenal medulla have also been shown to contribute to hyperalgesia in some stress models [7]. It should also be noted that ATP and other endogenous nucleotides, including diadenosine polyphosphates (Ap_4_A, Ap_5_A and Ap_6_A), are co-stored and co-released with catecholamines from adrenomedullary chromaffin cells [8,9,10]. Once released, ATP and adenosine nucleotides can regulate catecholamine release in a paracrine manner [11,12,13,14,15,16] but a long-distance action on peripheral tissues has also been proposed [17].

Prolonged stress (cold stress, immobilization, congestive heart failure, etc.) gives rise to a variety of functional adaptations at the adrenal medulla level (e.g., denser splanchnic nerve innervation, more efficient intercellular coupling, increased expression of α9 subunit-containing (α9*) nicotinic acetylcholine receptors (nAChRs), etc.), which translate into increased frequency of spontaneous synaptic currents and action potential firing, ultimately favoring chromaffin granule exocytosis and catecholamine release [18,19,20,21,22,23].

Here we have investigated whether neuropathic pain might act as a stressor, thereby inducing adaptive changes in the adrenal medulla, and also whether plasticity characteristic of peripheral sensitization of nociceptive neurons could also be observed in adrenomedullary chromaffin cells from rats with neuropathic pain. We have made use of chronic constriction injury (CCI) of the sciatic nerve as a model of neuropathic pain and evaluated the primary events involved in stimulus-secretion coupling from chromaffin cells. Our results show increased synaptic strength at the splanchnic nerve–chromaffin cell junction and augmented Ca^2+^-evoked exocytosis, which would facilitate the release of catecholamines and adenosine nucleotides. Likewise, chromaffin cells from neuropathic animals overexpress P2X3 and P2X7 receptors as well as transient receptor potential vanilloid-1 (TRPV1) ion channels, the classical polymodal nocisensors. This suggests that receptor plasticity reminiscent of peripheral sensitization of DRG cells develops in adrenomedullary chromaffin cells, which could, in turn, contribute to the expression of nocifensive behaviors.

## 2. Results

### 2.1. CCI Animals Develop a Painful Purinergic Tone Involving P2X3 Receptors

**Figure 1:**
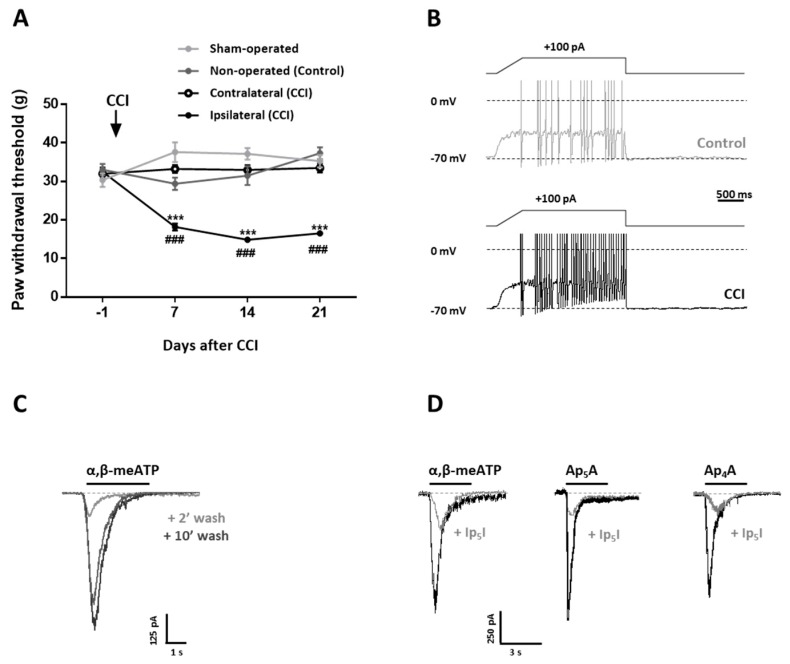
Dorsal root ganglia (DRG) neurons from chronic constriction injury (CCI) animals exhibit increased electrical excitability and P2X3 receptor-mediated currents. (**A**) CCI animals develop mechanical allodynia. Data show paw withdrawal thresholds to mechanical stimulation of the hindpaw’s plantar surface in the ipsilateral and contralateral paws from CCI animals (*n* = 16 rats), ipsilateral paw from sham-operated (*n* = 9 rats) animals, and both paws from non-operated (Control; *n* = 11 rats) animals. Behavioral testing was performed 1 day before (−1), 7, 14 and 21 days after CCI surgery. Statistical analysis was performed by a two-way ANOVA for repeated measures followed by a Bonferroni test to compare responses obtained at the same times (***, *p* < 0.001). Statistical significance of the effect of CCI with respect to baseline (−1) was assessed by a Student t test for paired data. (^###^, *p* < 0.001). (**B**) Current-clamp recordings of action potentials evoked by current injection in DRG neurons from control (non-operated) and CCI animals. Current protocol is shown at the top of the panel. V_comm_ = −70 mV. Results are representative of those obtained in 3 cells from Control or CCI animals, respectively. (**C**) Voltage-clamp recordings of currents evoked by α,β-methylene ATP (α,β-meATP) in DRG neurons. The horizontal bar shows the application of α,β-meATP (10 μM, 3 s), which was applied three times with 2 (+2’ wash) and 10 (+10’ wash) min intervals. Currents rapidly decayed in the presence of the agonist and required a minimum of 10 min washout for complete recovery from desensitization. (**D**) Sensitivity to diinosine pentaphosphate (Ip_5_I) of currents evoked by α,β-meATP, Ap_5_A or Ap_4_A in DRG neurons from Control animals. Horizontal bars show application of α,β-meATP (10 μM, 3 s), and the diadenosine polyphosphates, Ap_5_A (10 μM, 3 s) or Ap_4_A (10 μM, 3 s), which were applied twice each, at 10 min intervals in the absence or the presence of Ip_5_I (+Ip_5_I; 10 μM, 2 min). Results are representative of those obtained in 6, 8 and 8 cells with α,β-meATP, Ap_5_A or Ap_4_A, respectively.

CCI animals characteristically developed an increased sensitivity to mechanical stimulation in the hindpaw ipsilateral to nerve injury. This sensitization was observed 7 days after surgery and remained stable for the following two weeks. There were significant and consistent differences in nocifensive responses of the affected paw compared to pre-surgery baseline, the contralateral paw, as well in comparison to sham and non-operated animals (Figure 1A). This allowed us to choose non-operated animals as controls for both behavioral and in vitro experiments. Changes in nociceptive tests in neuropathic animals have been shown to correlate with greater electrical excitability of sensory DRG neurons, particularly those with unmyelinated axons (C fibers) and small somata (<30 µm in diameter) [5,24]. Patch-clamp recordings were made from small-to medium-sized DRG neurons to determine whether CCI altered their electrical excitability and expression of P2X3 receptors. The average diameter and membrane capacitance of Control (33.6 ± 2.8 µm and 36.0 ± 3.0 pF; *n* = 29) and CCI (31.8 ± 3.0 µm and 34.0 ± 3.2 pF; *n* = 12; *p* > 0.05) neurons were similar. The number of action potentials generated during a 2500-ms ramp-and-plateau depolarizing current injection was used as a measure of excitability in current-clamped cells (Figure 1B) [25]. By contrast, DRG neurons from CCI animals fired at a higher frequency than those from the Controls (19.4 ± 5.1 Hz, *n* = 3 vs. 8.2 ± 2.3 Hz, *n* = 3, respectively: *p* < 0.05).

In about 70% (22/31) of dorsal root ganglia (DRG) neurons, local application of α,β-methylene ATP (α,β-meATP) (10 μM), Ap_4_A (10 µM) or Ap_5_A (10 µM), three P2X3 receptor agonists [26,27], elicited rapidly activating and declining (<1 s) inward currents with peak amplitudes of 324 ± 90 pA (*n* = 6), 337 ± 46 pA (*n* = 8), and 383 ± 73 pA (*n* = 8), respectively (Figure 1C,D). Currents that display such kinetics have been suggested to be characteristic of those mediated by homomeric P2X3 receptors [28]. The remaining cells (9/31) displayed either slow or mixed (fast and slow) responses that were not further investigated since they have been attributed to heteromeric P2X2/3 receptors and a combination of P2X3 and P2X2/3 receptors, respectively [29]. Fast-declining currents displayed prolonged desensitization after repeated applications of agonist, requiring 10 min wash for full recovery (Figure 1C). This was therefore the time interval chosen in experiments where the effect of diinosine pentaphosphate (Ip_5_I) (10 µM), a selective antagonist of homomeric P2X3 receptors [30], was assayed. Figure 1D shows that Ip_5_I, administered 2 min before and during agonist application, markedly inhibited peak currents evoked by α,β-meATP (62 ± 15 pA, *n* = 6; *p* < 0.001), Ap_4_A (29 ± 8 pA, *n* = 8; *p* < 0.001) or Ap_5_A (40 ± 15 pA, *n* = 8; *p* < 0.001), indicating the involvement of P2X3 receptors in the generation of the currents.

**Figure 2:**
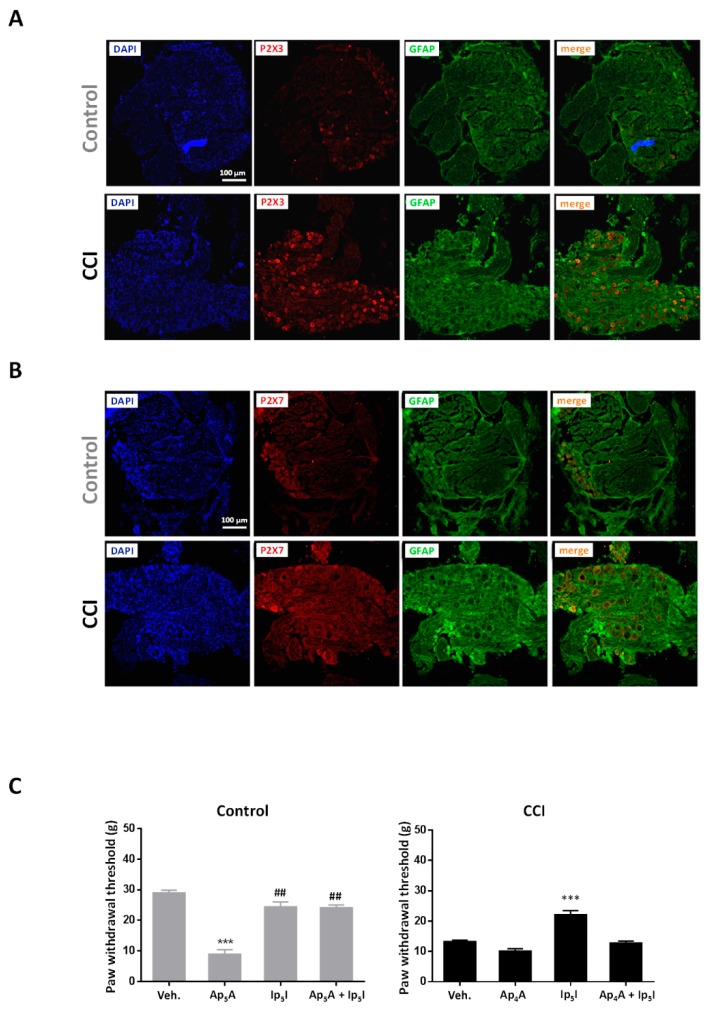
CCI animals show increased P2X3 and P2X7 receptor immunoreactivity in DRG and mechanical allodynia involving P2X3 receptors. (**A**) Fluorescence confocal images of DRG from control (upper panels) and CCI animals (lower panels) stained for P2X3 receptors and glial fibrillary acidic protein (GFAP). (**B**) Fluorescence confocal images of DRG from control (upper panels) and CCI animals (lower panels) stained for P2X7 receptors and GFAP. Cell nuclei in A and B were visualized following incubation with 4′6-diamidino-2-phenylindole (DAPI). Images are representative of those obtained in DRG from 3 Control and 3 CCI animals. (**C**) Effect of intraplantar (i.pl.) administration of diadenosine polyphosphates on paw withdrawal threshold to mechanical stimulation. **Left**; Effect of i.pl. administration of Ap_5_A (2 µg; *n* = 6 experiments, 3 rats), Ip_5_I (2 µg; *n* = 8 experiments, 4 rats), and co-administration of Ap_5_A and Ip_5_I (*n* = 6 experiments, 3 rats) in the hindpaw of control animals. **Right**; Effect of i.pl. administration of Ap_4_A (2 µg; *n* = 3 experiments, 3 rats), Ip_5_I (2 µg; *n* = 8 experiments, 4 rats), and co-administration of Ap_4_A and Ip_5_I (*n* = 4 experiments, 4 rats) in the ipsilateral hindpaw of CCI animals. Statistical significance was assessed by a Student t test for unpaired data with respect to vehicle (Veh.; ***, *p* < 0.001), and for paired data with respect to AP_5_A (^##^, *p* < 0.01).

Immunohistochemical experiments confirmed the expression of P2X3 receptors in DRG neurons. In agreement with previous reports [31,32,33], P2X3 receptor immunoreactivity predominated in small-to medium, round-shaped cells and did not overlap with that of glial fibrillary acidic protein (GFAP), a marker of satellite glial cells (SGC), indicating that labeled P2X3 receptors were expressed by DRG neurons and not glial cells (Figure 2A). In contrast, P2X7 receptor immunoreactivity was found to colocalize with GFAP, which indicates that the expression of this receptor is restricted to SGC (Figure 2B) [33,34]). Importantly, there was an increase in the number of cells positive for P2X3 and P2X7 and their labeling was more intense in DRG neurons from CCI animals than from the Controls, suggesting that both types of receptors are overexpressed following peripheral nerve injury (Figure 2A,B) [35,36,37].

Involvement of P2X3 receptors in pain signaling was evidenced by the ability of P2X3 agonists (Ap_4_A, Ap_5_A) and antagonist (Ip_5_I) to affect mechanical sensitivity in control and CCI animals (Figure 2C). Intraplantar (i.pl.) administration of Ap_5_A (2 µg) into the right hindpaw of control animals reduced the paw withdrawal threshold (PWT) to mechanical stimulation (from 28.9 ± 0.9 g to 8.9 ± 1.4 g, *n* = 6 experiments, 3 rats; *p* < 0.001 vs. vehicle). This effect was prevented when Ip_5_I (2 µg) was coinjected with Ap_5_A (24.2 ± 0.9 g, *n* = 6 experiments, 3 rats; *p* < 0.001 vs. Ap_5_A) while Ip_5_I administered alone was devoid of effect (24.4 ± 1.6 g, *n* = 8 experiments, 4 rats). These results indicate that homomeric P2X3 receptors mediate nociceptive responses in the periphery. At variance, i.pl. administration of Ip_5_I (2 µg) into the injured hindpaw of CCI animals increased the PWT (from 13.3 ± 0.4 g to 22.2 ± 1.3 g, *n* = 8 experiments, 4 rats; *p* < 0.001 vs. vehicle), hence suggesting the existence of a purinergic tone contributing to mechanical allodynia in CCI animals (Figure 2C). Importantly, the antiallodynic effect of Ip_5_I was reversed by coinjection of Ap_4_A (2 µg) (12.8 ± 0.6 g, *n* = 4 experiments, 4 rats; *p* < 0.001 vs. Ip_5_I), which on its own did not modify PWT values (10.1 ± 0.8 g, *n* = 3 experiments, 3 rats). These results suggest that homomeric P2X3 receptors are involved in the enhanced behavioral responses of CCI animals and are consistent with their overexpression in DRG neurons from these same animals [38].

### 2.2. Excitation-Secretion Coupling Plasticity in the Adrenal Medulla from CCI Animals

We investigated whether neuropathic pain might induce structural and functional adaptations in the adrenal medulla potentially leading to augmented catecholamine and nucleotide release. In particular, we analyzed changes in the sequence of events commonly referred to as “excitation-secretion coupling”, which commences when acetylcholine (ACh) molecules released from splanchnic nerve terminals bind to nAChRs in the membrane of chromaffin cells. Activation of nAChRs gives rise to excitatory postsynaptic potentials and action potential firing. Ca^2+^ entry during action potentials triggers the exocytotic release of the content of chromaffin granules.

**Figure 3:**
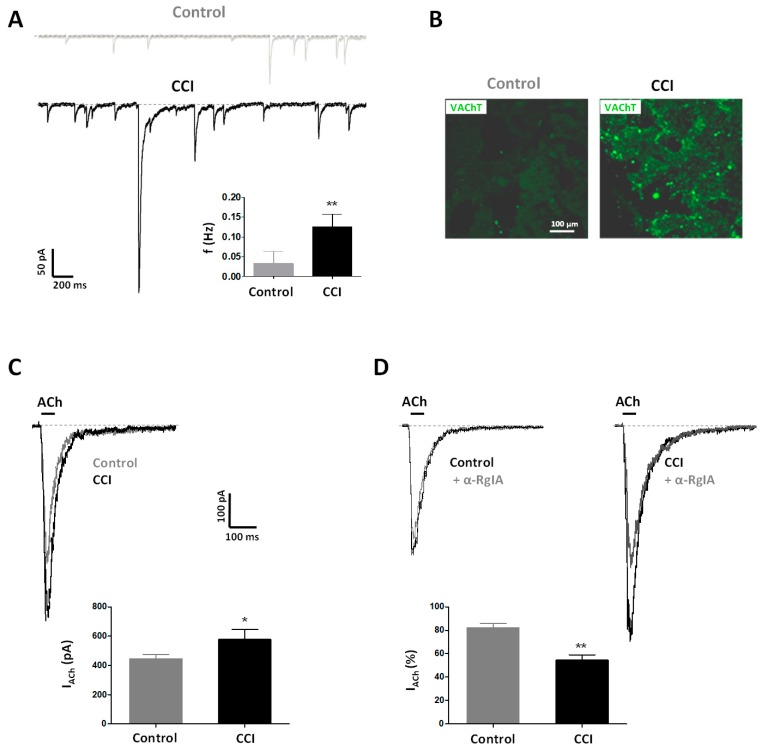
CCI animals show functional and structural remodeling of the splanchnic nerve–chromaffin cell junction. (**A**) sEPSCs recorded in chromaffin cells in tissue slices of the adrenal gland from Control and CCI animals. V_h_ = −80 mV. *Inset*. sEPSC frequency from Control (*n* = 6 cells, 3 rats) and CCI (*n* = 3 cells, 3 rats) animals. Statistical significance was assessed by a Student t test for unpaired data (**, *p* < 0.01). (**B**) Fluorescence confocal images of adrenal glands from Control and CCI animals stained for the vesicular acetylcholine transporter (VAChT). Images are representative of those obtained in 6 Control and 4 CCI animals. (**C**) Currents evoked by acetylcholine (ACh) in adrenal chromaffin cells from Control and CCI animals. ACh (100 µM) was applied during the time (50 ms) indicated by the horizontal bar. V_h_ = −80 mV. *Inset*. Peak amplitudes of currents evoked by ACh in chromaffin cells from Control (*n* = 66 cells, 19 rats) and CCI (*n* = 31 cells, 8 rats) animals. (**D**) Effect of α-RgIA on ACh-evoked currents in chromaffin cells from Control and CCI animals. ACh (100 µM, 50 ms) was applied as indicated by the horizontal bar in the absence and the presence of α-RgIA (+ α-RgIA, 200 nM, 2 min). V_h_ = −80 mV. *Inset*. Percentage of ACh-evoked currents remaining in the presence of α-RgIA from Control (*n* = 20 cells, 6 rats) and CCI (*n* = 4 cells, 1 rat) animals. Statistical significances were assessed by a Student t test for unpaired data (*, *p* < 0.05; **, *p* < 0.01). Scale bars are the same for panels (**C**,**D**).

Spontaneous excitatory postsynaptic currents (sEPSCs) were often observed in voltage-clamped chromaffin cells in adrenal slices from CCI animals (3 out of 3 cells), whereas they were almost absent in preparations from Control animals (6 out of 70 cells). Moreover, recordings obtained from CCI animals showed a higher frequency of sEPSCs (0.13 ± 0.03 Hz; *n* = 3 cells, 3 rats) than those from Control ones (0.04 ± 0.03 Hz; *n* = 6 cells, 3 rats; *p* < 0.01) (Figure 3A). These data are in line with a stronger staining of the vesicular transporter of acetylcholine (VAChT) in tissue slices from CCI animals (Figure 3B). Altogether, and in accordance with previous results on cold-stress rats [39], our results are suggestive of a higher density of splanchnic nerve terminals probably making synapses with chromaffin cells and, hence, contributing to the observed higher spontaneous synaptic activity in CCI animals.

Neuropathic pain was also associated with postsynaptic changes manifested by enlarged nicotinic currents evoked by exogenous application of ACh (100 µM, 50 ms) (Figure 3C). The peak amplitudes of nicotinic currents in chromaffin cells from CCI animals were 590 ± 24 pA (*n* = 31 cells, 8 rats) while currents recorded from Controls peaked at 453 ± 27 pA (n = 66 cells, 19 rats; *p* < 0.05). Interestingly, the fraction of the nicotinic current sensitive to α-conotoxin RgIA (α-RgIA; 200 nM), a selective blocker of α9* nAChRs [40,41], was greater in CCI animals (45 ± 4%; *n* = 4 cells, 1 rat) than in Control ones (17 ± 3%; *n* = 20 cells, 6 rats; *p* < 0.01), which indicates that the expression α9* nAChRs in chromaffin cells increases in conditions of neuropathic pain (Figure 3D).

**Figure 4:**
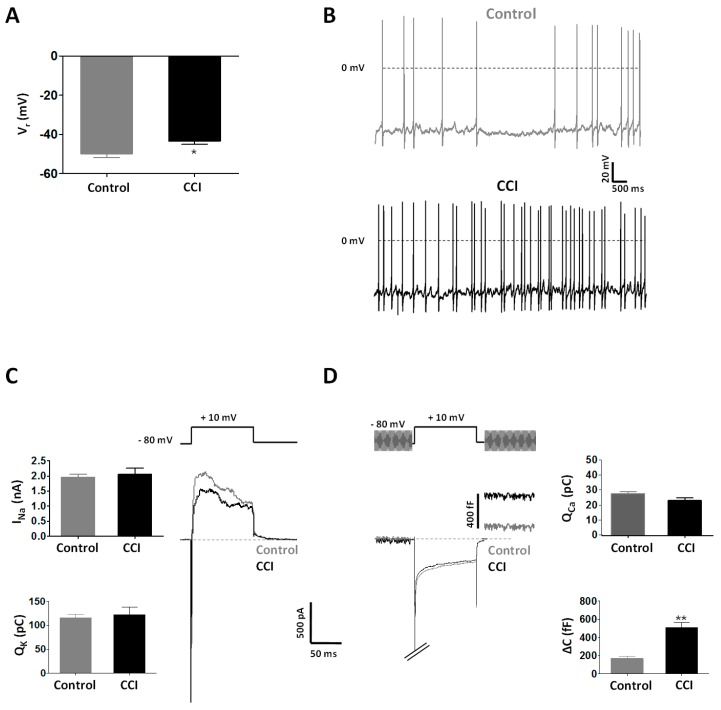
Chromaffin cells from CCI animals exhibit increased electrical excitability and exocytosis. (**A**) Resting membrane potential (V_r_) in chromaffin cells of the adrenal gland from Control (*n* = 40 cells, 17 rats) and CCI (*n* = 29 cells, 9 rats) animals. (**B**) Spontaneous action potentials recorded in chromaffin cells from Control and CCI animals. Recordings are representative of those obtained in 11 cells from 5 Control animals and 5 cells from 2 CCI animals, respectively. V_comm_ = −60 mV. (**C**) Voltage-activated Na^+^ and K^+^ currents in chromaffin cells from Control and CCI animals. The voltage protocol appears on top of the recordings. V_h_ = −80 mV. *Inset*. Peak amplitudes of Na^+^ currents (upper graph) and charge (Q) of K^+^ currents (lower graph) evoked by voltage depolarization in chromaffin cells from Control (*n* = 40 cells, 9 rats) and CCI (*n* = 29 cells, 9 rats) animals. (**D**) Voltage-activated Ca^2+^ currents and membrane capacitance changes in chromaffin cells from Control and CCI animals. The voltage protocol appears on top of the recordings. Note that membrane capacitance measurements were interrupted during membrane depolarization activating the Ca^2+^ conductance. V_h_ = −80 mV. *Inset*. Charge (Q) of Ca^2+^ current (upper graph) and increases in membrane capacitance (∆C; lower graph) evoked by voltage depolarization in chromaffin cells from Control (*n* = 25 cells, 9 rats) and CCI (*n* = 25 cells, 10 rats) animals. Statistical significances were assessed by a Student t test for unpaired data. *, *p* < 0.05; **, *p* < 0.01. Scale bars apply to panels (**C**,**D**).

Resting membrane potential (V_r_) was less negative in chromaffin cells from CCI animals (−43.5 ± 1.6 mV; *n* = 29 cells, 9 rats) than from Control ones (−50.0 ± 1.8 mV; *n* = 40 cells, 9 rats; *p* < 0.05) (Figure 4A). This possibly contributed to a higher frequency of spontaneous action potentials in cells from injured animals (1.7 ± 0.5 Hz; *n* = 5 cells, 2 rats) than from uninjured ones (1.1 ± 0.5 Hz; *n* = 11 cells, 5 rats; *p* < 0.01) (Figure 4B). Interestingly, no significant differences were observed in the amplitude of the main ionic currents (voltage-gated Na^+^, K^+^ and Ca^2+^ currents) involved in action potential generation as well as in membrane capacitance of chromaffin cells between the two experimental groups (Figure 4C,D). We also measured changes in membrane capacitance as an assay of exocytosis. Importantly, despite having similar voltage-gated Ca^2+^ entry (Q_Ca_ of 23.47 ± 1.77 pC, *n* = 25 cells, 10 CCI rats vs. 28.09 ± 1.52 pC, *n* = 25 cells, 9 Control rats), membrane capacitance increases were significantly larger in chromaffin cells from CCI animals (509 ± 55 fF; *n* = 9; 2 rats) than from Control ones (168 ± 23 fF; *n* = 5; 2 rats; *p* < 0.001) (Figure 4D). This result suggests a more efficacious organization of the secretory apparatus in chromaffin cells in conditions of neuropathic pain.

### 2.3. P2X3 Receptor Plasticity in Chromaffin Cells from CCI Animals

**Figure 5:**
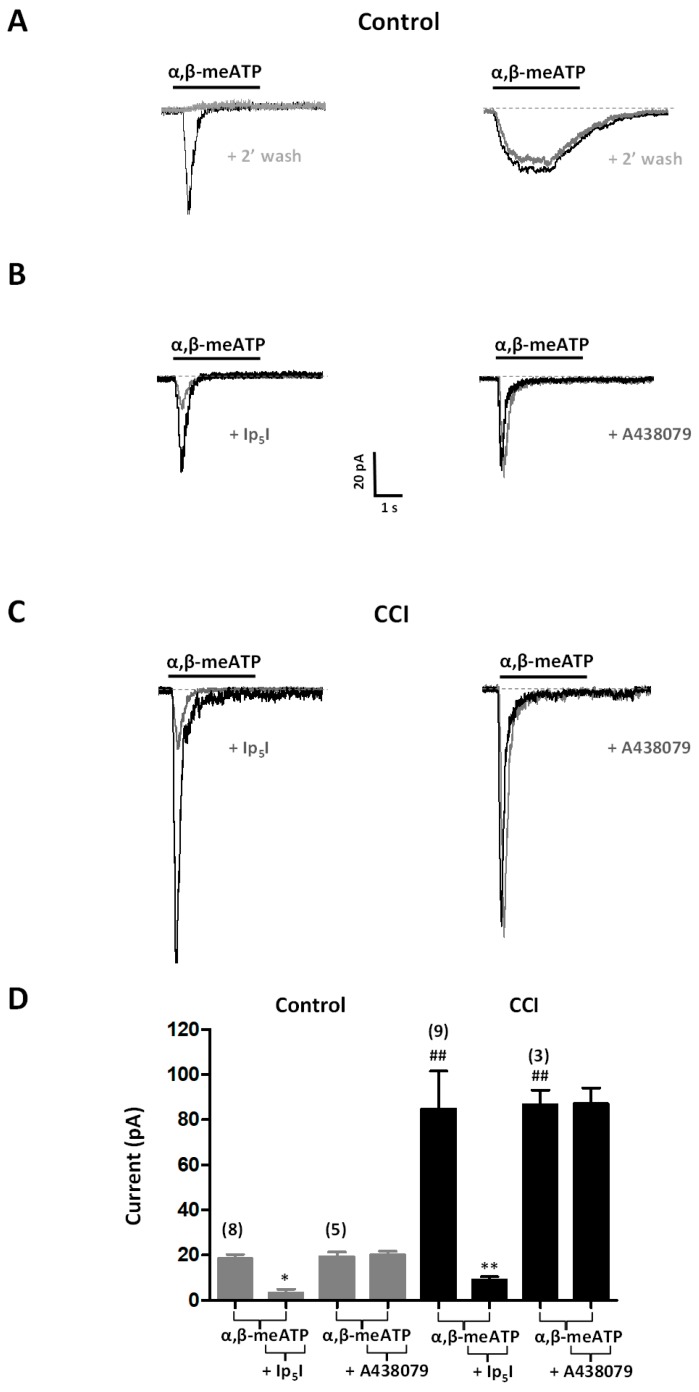
Functional plasticity of P2X3 receptors in chromaffin cells from CCI animals. (**A**) Voltage-clamp recordings of currents evoked by α,β-meATP in chromaffin cells of the adrenal gland from Control animals. The horizontal bars show the application of α,β-meATP (10 μM, 3 s), which was applied twice with a 2 min interval (+2’ wash). Representative recordings of rapidly desensitizing (left panel) and non-desensitizing (right panel) currents. (**B**) Sensitivity to Ip_5_I or to A438079 (a selective P2X7 receptor blocker) of currents evoked by α,β-meATP in chromaffin cells from Control animals. Horizontal bars show application of α,β-meATP (10 μM, 3 s), which was applied twice with a 10 min interval, in the absence or the presence of Ip_5_I (+Ip_5_I; 10 μM, 2 min; left panel) or A438079 (+A438079; 10 μM, 2 min; right panel). (**C**) Similar to B but referred to CCI animals. Scale bars apply to panels (**A**–**C**). (**D**). Amplitudes of 10 µM α,β-meATP-evoked currents in chromaffin cells from Control and CCI animals in the absence and the presence of Ip_5_I (+Ip_5_I; 10 μM) or A438079 ((+A438079; 10 μM). Data from the number of cells are shown between parentheses on top of bars. Statistical significances were assessed by a Student *t* test for paired data when evaluating the effect of receptor antagonists (*, *p* < 0.05; **, *p* < 0.01), and for unpaired data when evaluating the effect of CCI (^##^, *p* < 0.01).

We also used the patch-clamp technique to characterize functional P2X3 receptors in chromaffin cells in tissue slices from the adrenal gland. Current responses to P2X3 receptor agonists (α,β-meATP and Ap_4_A) locally applied to the cell under investigation were compared between Control and CCI preparations. α,β-meATP (10 µM, 3 s) induced inwardly-directed currents in 37% (28 out of 76 cells) of cells tested from Control animals (*n* = 5 rats). Interestingly, current responses differed in their inactivation kinetics; 89% of responding cells (25 out of 28) showed fast inactivating responses, whereas the other 11% displayed sustained, non-inactivating currents. Moreover, sustained currents could be reproducibly elicited by α,β-meATP, applied at 2 min intervals, while the fast-inactivating ones could not (Figure 5A). Ap_4_A (10 µM, 3 s) also activated currents in 36% of cells tested (5 out of 14 cells; *n* = 2 rats), all which were fast declining (data not shown). Due to the low occurrence of non-desensitizing currents and our interest in homomeric P2X3 receptors, we focused our efforts on the characterization of transient currents. Importantly, the semisynthetic dinucleotide Ip_5_I (10 µM) markedly reduced fast-inactivating currents evoked by α,β-meATP (80% reduction, *n* = 8 cells; 2 rats) (Figure 5B,D) and Ap_4_A (60% reduction, *n* = 3 cells; 1 rat) (data not shown).

In preparations from CCI animals, the percentage of cells responding to α,β-meATP or Ap_4_A rose to 81% (21 out of 26 cells; *n* = 4 rats) and 100% (13 out of 13 cells; *n* = 3 rats), respectively. The relative proportion of cells responding to α,β-meATP with fast inactivating currents did not significantly change in preparations from CCI animals with respect to those from Control animals. It is also noteworthy that the amplitudes of the currents evoked by α,β-meATP (44 ± 9 pA; *n* = 19 cells) and Ap_4_A (56 ± 6 pA; *n* = 13 cells) were larger in CCI animals as compared to Control ones (28 ± 9 pA; *n* = 25 cells for α,β-meATP, and 9 ± 1 pA; *n* = 5 cells for Ap_4_A; *p* < 0.01 for both agonists) (Figure 5B–D). α,β-meATP- and Ap_4_A-evoked currents in CCI animals were inhibited by Ip_5_I (10 µM) to a similar extent (89%, *n* = 9 cells; 3 rats in the case of α,β-meATP, and 88%, *n* = 3 cells; 1 rat in the case of Ap_4_A) than in Control animals. As an additional test of the selectivity of α,β-meATP, we assayed the effect of A438079, a selective P2X7 receptor blocker, on α,β-meATP-evoked currents. No effect of A438079 (10 µM) could be observed, irrespective of the source, Control or CCI animals, of the tissue slices, hence indicating that α,β-meATP does not activate P2X7 receptors in chromaffin cells (Figure 5B–D).

**Figure 6:**
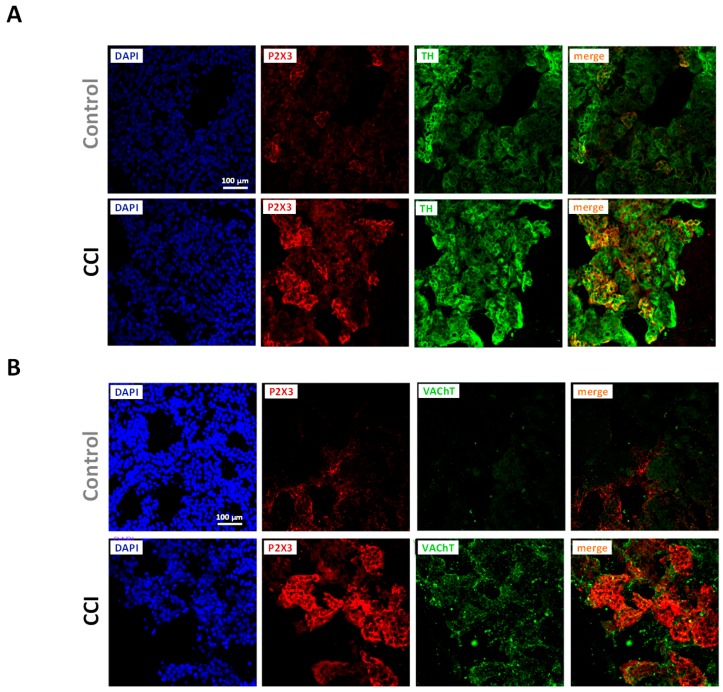
Increased P2X3 receptor immunoreactivity in chromaffin cells from CCI animals. (**A**) Fluorescence confocal images of adrenal glands from Control (upper panels) and CCI animals (lower panels) stained for P2X3 receptor and tyrosine hydroxylase (TH). (**B**) Fluorescence confocal images of adrenal glands from Control (upper panels) and CCI animals (lower panels) stained for P2X3 receptors and the vesicular acetylcholine transporter (VAChT). Cell nuclei in (**A**,**B**) were visualized following incubation with DAPI. Images are representative of those obtained from 3 Control and 3 CCI animals.

In agreement with functional results, immunohistochemistry confirmed the expression of P2X3 receptors in the adrenal medulla. The lobular pattern of labeling and colocalization with tyrosine hydroxylase (TH), an enzyme involved in catecholamine biosynthesis, indicate that P2X3 receptors are located in chromaffin cells; in contrast, no colocalization was observed between P2X3 and VAChT immunoreactivities, hence suggesting that P2X3 expression may be restricted to chromaffin cells. Likewise, the higher percentage of cells responding to P2X3 agonists was paralleled by more intense staining as well as to a broader distribution of P2X3 immunoreactivity in adrenals from CCI animals (Figure 6A,B).

### 2.4. P2X7 Receptor Plasticity in Chromaffin Cells from CCI Animals

**Figure 7:**
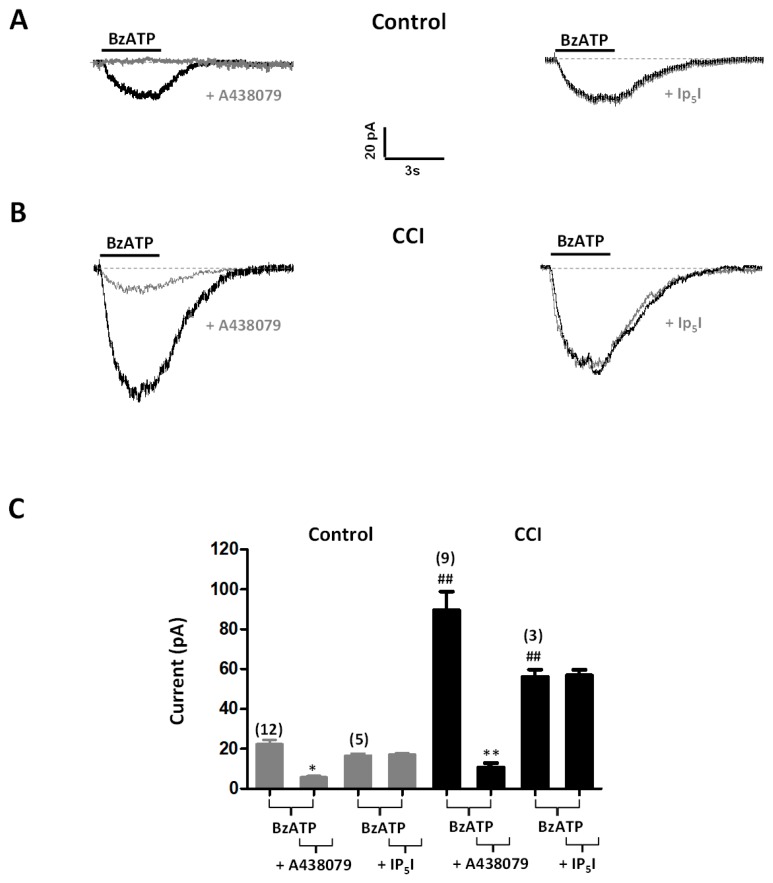
Functional plasticity of P2X7 receptors in chromaffin cells from CCI animals. (**A**) Voltage-clamp recordings of currents evoked by BzATP (a P2X7 receptor agonist) in chromaffin cells of the adrenal gland from Control animals. The horizontal bars show the application of BzATP (100 μM, 3 s), which was applied twice with a 10 min interval in the absence or the presence of A438079 (+A438079; 10 μM, 2 min; left panel) or of Ip_5_I (+Ip_5_I; 10 μM, 2 min; right panel). (**B**) Similar to panel (**A**), but referred to CCI animals. Scale bars apply to panels (**A**,**B**). (**C**) Amplitudes of 100 µM BzATP-evoked currents in chromaffin cells from Control and CCI animals in the absence and the presence of Ip_5_I ((+Ip_5_I; 10 μM) or A438079 (+A438079; 10 μM). Data from the number of cells are shown between parentheses on top of bars. Statistical significances were assessed by a Student *t* test for paired data when evaluating the effect of receptor antagonists (*, *p* < 0.05; **, *p* < 0.01), and unpaired data when evaluating the effect of CCI (^##^, *p* < 0.01).

Current responses to BzATP (100 µM; 3 s), a P2X7 receptor agonist, were observed in 74% (14 out of 19 cells; 4 rats) and 86% (19 out of 22 cells; *n* = 7 rats) of chromaffin cells from Control and CCI animals, respectively. Currents rose over the 3 s of agonist application in accordance with the lack of desensitization of P2X7 receptors. Likewise, peak amplitudes were significantly larger in cells from CCI animals (86.89 ± 16.00 pA, *n* = 19 cells) than from Control ones (23.49 ± 3.23 pA, *n* = 14 cells; *p* < 0.01) (Figure 7A–C). BzATP-evoked currents were inhibited by A438079 (10 µM). Percent inhibition was similar in control (74%, *n* = 12 cells; 2 rats) and CCI (88%, *n* = 9 cells; 2 rats) animals. By contrast, no inhibitory effect was observed in the presence of Ip_5_I (10 µM) in tissue slices from either Control or CCI animals (Figure 7A–C), thus implying that BzATP does not activate P2X3 receptors in chromaffin cells. 

**Figure 8:**
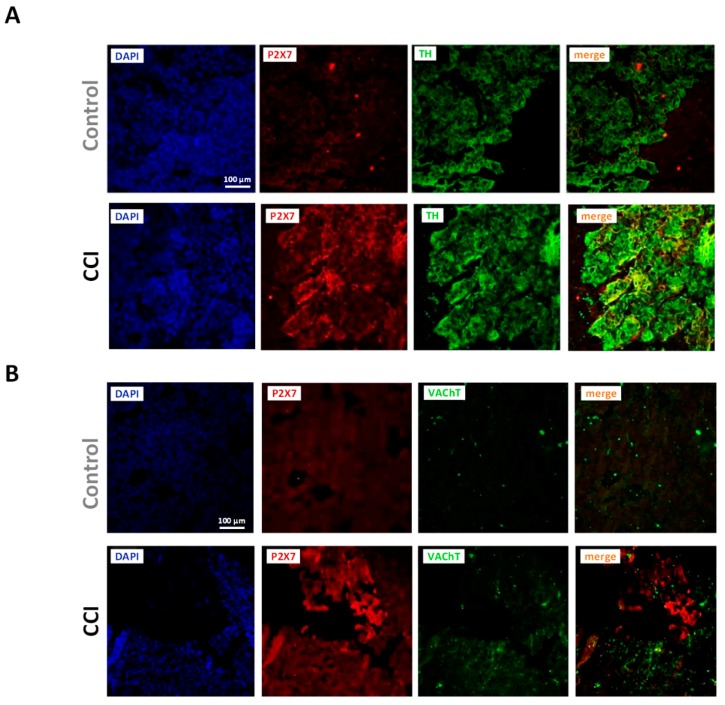
Increased P2X7 receptor immunoreactivity in chromaffin cells from CCI animals. (**A**) Fluorescence confocal images of adrenal gland slices from Control (upper panels) and CCI animals (lower panels) stained for P2X7 receptor and tyrosine hydroxylase (TH). (**B**) Fluorescence confocal images of adrenal gland slices from Control (upper panels) and CCI animals (lower panels) stained for P2X7 receptor and the vesicular acetylcholine transporter (VAChT). Cell nuclei in (**A**,**B**) were visualized following incubation with DAPI. Images are representative of those obtained from 3 Control and 3 CCI animals.

Immunohistochemistry confirmed the expression of P2X7 receptors in the rat adrenal medulla. P2X7 receptor labeling was diffuse, extending along the medullary part of the slice. Clear differences in intensity were observed between Control and CCI preparations. P2X7 receptor and TH immunostaining overlapped, hence suggesting that P2X7 receptors are localized in chromaffin cells; at variance, VAChT and P2X7 immunoreactivity appear, for their most part, clearly separated (Figure 8A,B).

### 2.5. TRPV1 Channel Plasticity in Chromaffin Cells from CCI Animals

**Figure 9:**
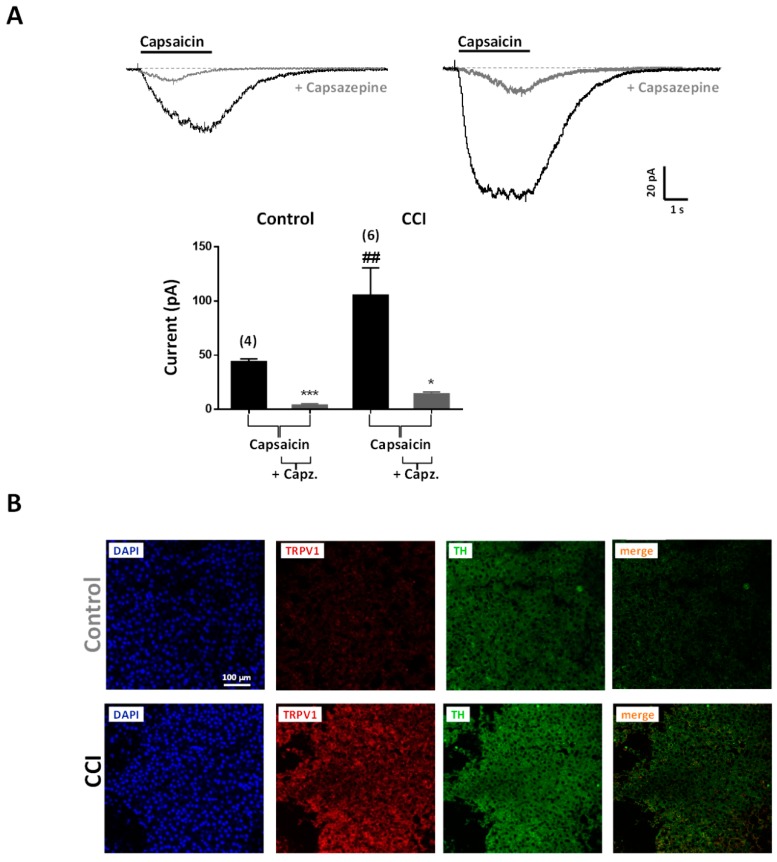
Plasticity of TRPV1 channels in chromaffin cells from CCI animals. (**A**) Voltage-clamp recordings of currents evoked by capsaicin in chromaffin cells of the adrenal gland from Control (left part) and CCI (right part) animals. The horizontal bars show the application of capsaicin (10 μM, 3 s), which was applied twice with a 10 min interval in the absence or the presence of capsazepine (+ Capsazepine; 10 μM, 2 min). *Inset*. Amplitudes of 10 µM capsaicin-evoked currents in chromaffin cells from Control and CCI animals in the absence and the presence of capsazepine (Capz.; 10 μM). Data are from the number of cells and animals shown between parentheses on top of bars. Statistical significances were assessed by a Student *t* test for paired data when evaluating the effect of capsazepine (*, *p* < 0.05; ***, *p* < 0.001), and unpaired data when evaluating the effect of CCI (^##^, <0.01). (**B**) Fluorescence confocal images of adrenal gland slices from Control (upper panels) and CCI animals (lower panels) stained for TRPV1 channels and tyrosine hydroxylase (TH). Cell nuclei were visualized following incubation with DAPI. Images are representative of those obtained from 3 Control and 3 CCI animals.

The expression of TRPV1 channels was also investigated in chromaffin cells from the adrenal medulla. TRPV1 channels are non-selective cation channels with high permeability to Ca^2+^ that are receptive to noxious heat (above 43 °C), capsaicin, endovanilloids, and low pH [42,43]. Capsaicin (10 μM) induced current responses in 23% (7 out of 30 cells) of cells from Control animals (*n* = 3 rats) and in 70% (30 out of 43 cells) of the cells tested from CCI animals (*n* = 5 rats). In addition, currents were of greater amplitude in CCI animals (105.3 ± 2.2 pA; *n* = 30) than in Control ones (44.0 ± 2.5 pA; *n* = 7; *p* < 0.001) and markedly sensitive to the selective antagonist capsazepine (10 μM, *n* = 6 and 4 cells, respectively) (Figure 9A). In agreement with functional results, immunohistochemical data showed enhanced labeling of TRPV1 channels in the adrenal medulla of the CCI animals with respect to control ones. The immunoreactivity of TRPV1 channels showed a broad distribution, similar to that of TH, which suggests their localization in chromaffin cells (Figure 9B).

## 3. Discussion

The results of three distinct sets of experiments have been obtained in the present work: (1) CCI of the rat’s sciatic nerve overexpresses P2X3 and P2X7 receptors in DRG cells that could contribute to mechanical hypersensitivity in the affected hindpaw; (2) the adrenal medulla from CCI animals undergoes functional and structural modifications similar to those reported in a model of prolonged cold-stress. Such modifications could lead to a higher output of catecholamines and adenosine nucleotides able to regulate chromaffin cell function locally and also to exert a systemic action; and (3) in chromaffin cells from neuropathic animals, there is an increased expression of functional P2X3 and P2X7 receptors as well as TRPV1 channels, reminiscent to that observed in the peripheral nociceptive system.

### 3.1. P2X3 and P2X7 Receptors Are Overexpressed in DRG from CCI Animals

In agreement with previous data, CCI animals developed mechanical allodynia in the plantar surface of the hindpaw ipsilateral to nerve injury [44]. Nocifensive behavior persisted over 2 weeks, which allowed stable testing of the antiallodynic effect of investigational drugs; likewise, these compounds can be assayed in a variety of control conditions (contralateral hindpaw in CCI animals, sham-operated hindpaw, and hindpaws of non-operated animals) to test for potential algesic or analgesic properties. In behavioral tests, we have employed three dinucleotides, Ap_4_A, Ap_5_A and Ip_5_I, each one having specific features. Ap_4_A and Ap_5_A are naturally occurring adenosine dinucleotides with full agonist activity on P2X3 receptors but different efficacies for P2X2 receptors. Specifically, Ap_4_A is a full agonist of P2X2 receptors, whereas Ap_5_A is inactive [27]. Ip_5_I is a semisynthetic inosine dinucleotide that behaves as an effective antagonist at rat homomeric P2X3 receptors (IC_50_ of ≈3 µM) but not at heteromeric P2X2/3 receptors [28,30]. Our results confirmed the involvement of homomeric P2X3 receptors in mechanical sensitivity in both Control (non-operated) and CCI animals. In particular, i.pl. administration of Ap_5_A induced mechanical allodynia in Control animals which was prevented by co-injection of Ip_5_I thus pointing to the involvement of homomeric P2X3 receptors. Interestingly, injection of Ip_5_I alone reduced mechanical sensitivity in CCI animals, this effect being occluded by the presence of Ap_4_A. This result implies the existence of a purinergic algesic tone in CCI animals, which is probably mediated by homomeric P2X3 receptors.

DRG neurons are known to express homomeric P2X3 receptors that display rapid decaying current kinetics with long-lasting desensitization [28,29,30]. We observed this type of responses in dissociated DRG neurons upon application of α,β-meATP, Ap_4_A or Ap_5_A, which were reduced in the presence of Ip_5_I (10 µM) thus demonstrating the existence of functional homomeric P2X3 receptors. Moreover, histological data corroborated the results of pharmacological experiments by localizing P2X3 immunoreactivity in DRG neurons. Importantly, DRG neurons from CCI animals showed increased electrical excitability to current injection as well as stronger P2X3 receptor immunoreactivity. Presumably, both an augmented depolarization elicited by P2X3 receptor activation and an easier action potential discharge may act synergistically to produce sensitization of nociceptors, which would contribute to the mechanical allodynia characteristic of neuropathic pain.

Lastly, immunohistochemistry also indicated that P2X7 receptor expression is restricted to SGC in DRG, as previously described [45]. Interestingly, plasticity of pain pathways in neuropathic conditions also involves these non-neuronal cells, which not only enwrap but also communicate with DRG neurons. P2X7 receptors were overexpressed in SGC from CCI animals, and therefore could contribute to intercellular communication in DRG by favoring ATP and proinflammatory interleukin-1β release, ultimately affecting afferent inputs into the spinal cord [36,46,47].

Altogether, these results recapitulate and extend previous data concerning the role of P2X3 and P2X7 receptors and of two endogenous diadenosine polyphosphates (Ap_4_A and Ap_5_A) in the CCI model of neuropathic pain. Furthermore, it will allow an adequate comparison with results obtained in the adrenal medulla.

### 3.2. Neuropathic Pain Reproduces Morphofunctional Changes Induced by Prolonged Stress in the Adrenal Medulla

Activation of the sympathoadrenal system is commonly implicated in the classic “fight or flight” response to acute stressors. To cope with stress, chromaffin cells exocytose the content of their granules. Nevertheless, sustaining adrenal medulla response over time implies the adaption of excitation-secretion coupling to increased body demands. The mechanisms by which the adrenal medulla maintains exocytosis during prolonged stress have been thoroughly investigated in a chronic cold-stress model in which animals are exposed to an ambient temperature of 4 °C for 5 days. Data reported from this model were obtained in tissue slices of the adrenal gland and revealed upregulation of chemical transmission at the splanchnic nerve-chromaffin cell synapse, overexpression of α9* nAChRs, increased chromaffin cell excitability, and enhanced gap junction-mediated communication between chromaffin cells [19,20,48].

Here, we have observed a higher frequency of sEPSCs in adrenal gland slices from CCI animals compared to Controls, possibly reflecting a denser cholinergic innervation as deduced from increased VAChT immunoreactivity. Also, postsynaptic nAChRs undergo functional plasticity since exogenous ACh evoked nicotinic currents with larger amplitudes. Furthermore, taking advantage of the specific blockade of α9* nAChRs by α-RgIA, we could show a larger contribution of this nAChR subtype to whole-cell currents in CCI animals. In addition, chromaffin cells from CCI animals exhibit slightly depolarized resting membrane potential and a more frequent discharge of spontaneous action potentials. All these changes might converge to improve stimulus-secretion coupling efficiency. Secretion efficiency can also be augmented through increased gap junction coupling between chromaffin cells, an issue that has not been explored in the current study, and through modifications in the secretory apparatus itself. In this respect, our capacitance measurements indicated increased exocytosis evoked by voltage-activated Ca^2+^ entry. Importantly, ATP is essential for the accumulation of catecholamines in chromaffin granules and for their exocytosis [49]. Chromaffin granules contain catecholamines at 0.8–1 M, ATP at ≈150 mM, Ap_4_A at 6 mM, and Ap_5_A at 6 mM in an acidic pH of ≈5.5 [50,51], and all are secreted simultaneously [8,10]. Clearly, further experiments need to be done to ascertain the mechanisms (changes in quantal size, quantal content, and probability of release) underlying such a facilitation of the secretory response.

Our results suggest that CCI may act as a stressor capable of inducing profound modifications in adrenal medulla function. Most likely, these modifications follow reflex effects on splanchnic nerve output triggered by spontaneous activity of DRG neurons or mechanical stimulation of the injured hindpaw [52]. On the other hand, it is difficult to envisage how enhanced secretory activity of chromaffin cells could be adaptive in a situation of neuropathy, unless it would tend to alleviate pain and restore normal nociception. In fact, there are numerous reports of stress-induced hyperalgesia and of the algesic effect of catecholamines [7,25,53,54] and endogenous adenosine nucleotides [1,55,56]). Elucidation of the impact of adrenal medulla activation on severity and time course of neuropathic pain was beyond the scope of the present study, but certainly will deserve a detailed investigation not only for a better understanding of the intricate relation between stress and pain but also to design more appropriate medical interventions targeting specific arms of the SNS.

### 3.3. Chromaffin Cells from CCI Animals Overexpress Functional P2X3 and P2X7 Receptors and TRPV1 Channels

We have characterized P2X3 and P2X7 receptors in adrenal gland slices by using the same experimental approach and reagents that allowed their identification in DRG cells. As was the case for DRG neurons, α,β-meATP elicited inward currents in chromaffin cells from Control animals with either rapid or non-decaying kinetics suggesting the expression of homomeric P2X3 receptors and/or heteromeric P2X2/3 receptors [30]. Transient currents were substantially reduced by Ip_5_I, indicating that they were mediated by homomeric P2X3 receptors. By contrast, A438079 left α,β-meATP-evoked currents unaffected thus excluding the involvement of P2X7 receptors. P2X3 receptors underwent functional plasticity in chromaffin cells from CCI animals as evidenced by larger Ip_5_I-sensitive currents evoked by α,β-meATP. Larger current amplitudes were associated with increased staining of P2X3 protein in chromaffin cells suggesting an upregulation of P2X3 receptors and is consistent with a larger number of cells exhibiting current responses to α,β-meATP in slices from CCI animals. Parallel results were obtained regarding the expression of P2X7 receptors. Currents evoked by BzATP in chromaffin cells were insensitive to Ip_5_I administration but were almost suppressed by A437089 [57]. Likewise, BzATP-activated currents activated slowly and desensitized slowly in accordance with currents mediated by P2X7 receptors in other cell types [58,59]. Immunohistochemistry confirmed the expression of P2X7 receptors in chromaffin cells as well as increased staining in adrenal slices from CCI animals. Consequently, bigger BzATP-activated currents were recorded in chromaffin cells from these animals.

Given that chromaffin cells share embryological origin with DRG neurons, we also looked for the presence of TRPV1 channels in chromaffin cells. Again, results from immunohistochemical and electrophysiological experiments concurred in pointing to the expression of functional TRPV1 channels mediating currents whose amplitude increased in CCI animals. The fact that the number of cells giving a detectable response to capsaicin also increased in neuropathic animals is also suggestive of an upregulation of TRPV1 channels. In this respect it is of note that P2X3 receptors and TRPV1 channels colocalize in small to medium size rat DRG neurons [60,61] and that expression of TRPV1 channels increases following CCI [62].

The existence of P2X receptors in the adrenal gland has been previously reported by immunohistochemical, molecular and functional assays. P2X2 receptors were originally cloned from rat pheochromocytoma cells, the tumor counterpart of chromaffin cells [63], and together with P2X1, P2X3, P2X5 and P2X7 receptors were identified by immunohistochemistry in chromaffin cells from the same species [64,65,66]. ATP-activated currents have been observed in subpopulations of cultured guinea-pig and cow chromaffin cells [67,68,69,70] but have not been recorded from rat cells [67,68,69,70]. In our hands, small responses to α,β-meATP were observed in a relatively small fraction (37%) of the cells in tissue slices from Control animals. It may well be that trypsin treatment reported during cell isolation could have eliminated P2X3 receptors in the plasma membrane of chromaffin cells [67,68,69,70]. In addition, time in culture and the age of the animals (17 days in [67,68,69,70], and ≈2 months in this study) are also known to change the expression of P2X3 receptors [66,70,71]. To the best of our knowledge, this is the first report of functional P2X7 receptors and TRPV1 channels in chromaffin cells.

The identification of P2X3 and P2X7 receptors and TRPV1 channels in chromaffin cells raises the question as to their physiological role. Purinergic P2X receptors can be activated in an autocrine or paracrine manner by adenosine nucleotides (ATP, Ap_4_A, Ap_5_A, etc.) released by exocytosis of chromaffin granules. We are tempted to hypothesize that P2X3 and P2X7 receptors activated by released adenosine nucleotides may briefly depolarize chromaffin cells to trigger action potentials, Ca^2+^ entry and exocytosis, particularly when those receptors are overexpressed in neuropathic animals [37,72]. It is also possible to speculate that TRPV1 channels can be activated or sensitized by a low pH, locally generated by the acidic (pH ≈ 5.5) content of exocytosed chromaffin granules [73,74]. Interestingly, a pH reduction also slows desensitization of homomeric P2X3 receptors. Simultaneous activation of P2X3 receptors and TRV1 channels might translate into increased secretory activity but might also favor cross-inhibition between those two ligand-gated channels perhaps to prevent overstimulation of chromaffin cells [75]. Further studies are certainly needed to clarify the exact role that concurrent activation of P2X3 and P2X7 receptors and TRPV1 channels might play in the control of adrenal medulla function.

## 4. Materials and Methods

Male Sprague–Dawley rats (160–200 g/6–8 weeks old) were used. Animals were housed in transparent cages at 23 °C and had free access to food and water. All procedures in this study were conducted according to the animal welfare guidelines of the European Community (project identification code BFU2011-26253) and were approved by the Committee on Animal Experimentation of the Universidad Complutense de Madrid (approval date, 1 June 2011).

### 4.1. Chronic Constriction Injury of the Sciatic Nerve

Rats underwent loose ligation of the right sciatic nerve as described previously by Bennett and Xie (1988) [76]. Surgical procedures were performed on the right paw under sterile conditions and ketamine (100 mg/kg; Merial Labs, Barcelona, Spain) and medetomidine (100 µg/kg; Dr. Esteve Labs, Barcelona, Spain) intraperitoneal anesthesia. Proximal to the sciatic trifurcation, the nerve (approximately 7 mm) was freed from adhering tissue and four loose ligatures (1 mm apart) barely constricting the nerve were tied around using 4/0 chromic catgut. In sham surgery, the nerve was exposed but was not ligated. The incision was closed in layers with silk thread 6/0.

### 4.2. Behavioral Testing of Mechanical Allodynia

On each day, rats were habituated to the experimental room for at least 30 min prior to testing. All behavioral tests were conducted between 09:00 and 12:00 h. PWT were determined for the right and left paws with an automated dynamic plantar aesthesiometer (2.5 g/s, cut-off 50 g; Ugo Basile, Gemonio, Italy) [77]. The nerve-injured (CCI) and sham-operated or non-operated paws (Control) were tested in each rat. Assessments were carried out prior to surgery (mean of 3 measurements on alternate days the week preceding surgery, designated as day −1) and on post-surgery days 7, 9, 11, 14, 17 and 21, when abnormal pain behavior was at a stable maximum. Each test was repeated 3 times at 5-min intervals and the mean value reported. Mechanical hypersensitivity was defined as at least a 25% decrease in PWT compared with pre-CCI baseline. Rats not exhibiting mechanical hypersensitivity were discarded. Responsiveness to mechanical stimuli was determined prior to (three times, 5 min apart) and at 30 min after i.pl. injection of saline (control responses) or purinergic drugs. Intraplantar injections were given in a volume of 20 µL using a Hamilton syringe with a 30G gauge needle. Coinjection of agonists and antagonists was done such that antagonists entered the paw first. This was accomplished by drawing up 10 µL of the antagonist, then drawing up a small amount of air into the syringe (to avoid mixing of drugs in the syringe) and finally 10 µL of the antagonist were drawn up into the syringe [25]. Each animal received up to three injections with a minimum separation of 48 h.

### 4.3. Isolation of DRG Neurons

Rats were sacrificed by cervical dislocation followed by decapitation and lumbar segments of the spinal column were removed and placed in a cold Ca^2+^, Mg^2+^-free Hank’s solution (Sigma-Aldrich, Madrid, Spain). The bone surrounding the spinal cord was removed and the L4, L5 and L6 DRG were exposed and pulled out (only from the right side in CCI animals). After removing the roots, DRG were chopped in half and incubated for 60 min at 37 °C in Dulbecco’s modified Eagle’s Medium-low glucose (DMEM; Sigma-Aldrich) containing 5 mg/mL collagenase XI (Worthington Biochemical, Lakewood, NJ, USA), 100 U/mL penicillin (Sigma-Aldrich), and 0.1 mg/mL streptomycin (Sigma-Aldrich). Then, the cell suspension was washed with DMEM by centrifugation (300 G, 5 min at 4 °C), filtered through a 100 µm mesh to eliminate cell clumps and washed again by centrifugation. The cell pellet was resuspended in DMEM and 40 µL were dropped onto 10 mm diameter glass coverslips treated with poly-d-lysine (1 mg/mL, 30 min; Sigma-Aldrich) placed in 35 mm diameter Petri dishes. Finally, plated cells were flooded with 2.5 mL of DMEM supplemented with 10% fetal calf serum (Sigma-Aldrich, Madrid, Spain), 100 U/mL penicillin and 0.1 mg/mL streptomycin, and stored in an incubator (Hera Cell, Heraeus, Hanau, Germany) with a 5% CO_2_/95% air atmosphere at 37 °C. This protocol yields spherical cell bodies without neurites, from which only small to medium DRG neurons (diameter <40 μm) [24], were chosen for recording within 12–24 h of plating.

### 4.4. Adrenal Gland Preparation

Acute tissue slices of the rat adrenal gland were prepared as previously described [20]. Briefly, adrenal glands were removed from rats that had been killed by decapitation after cervical dislocation. Both, CCI (7–21 days after surgery) and Control animals were used. After removal, the glands were kept in ice-cold saline, and one gland glued onto an agarose cube and placed on the stage of a vibratome (Integraslice 7550 MM, Campden Instruments, Loughborough, UK). Slices of 300 μm thickness (6–8 slices per gland) were cut with a razor blade and transferred to a storage chamber maintained at 37 °C, containing Ringer’s saline (in mM): 125 NaCl, 2.5 KCl, 2 CaCl_2_, 1 MgCl_2_, 1.25 NaH_2_PO_4_, 26 NaHCO_3_, and 12 glucose (pH 7.4 adjusted with HCl). The saline was continuously bubbled with carbogen (95% O_2_/5% CO_2_). Slices were subsequently transferred to a submersion chamber attached to the stage of an upright microscope (Olympus BX51W1, Barcelona Spain), fixed with a nylon grid and superfused with saline solution at room temperature. Cells were viewed under a 63x water immersion objective and a DL-604 OEM camera (Oxford Instruments, Abingdon, UK).

### 4.5. Electrophysiological Recordings

All electrophysiological recordings were performed in the perforated-patch variant of the whole-cell configuration of the patch-clamp technique with an EPC10 amplifier using PatchMaster software (HEKA Electronic, Lambrecht, Germany) [78]. Patch pipettes were pulled from borosilicate glass to have final resistance of 5.5–8.5 MΩ when filled with internal solution (see below). Membrane currents were filtered at 1 (sEPSCs and ligand-activated currents) or 3 (voltage-activated currents) kHz, and sampled at 10  kHz from cells held at a voltage (V_h_) of −80 mV. Series resistance (<20 MΩ) was compensated by 80% and monitored throughout the experiment together with the cell membrane capacitance. The quantity of charge Q was calculated as the time integral of the inward (Ca^2+^) or outward (K^+^) currents. Given the presence of an early inward Na^+^ current, the limits for the current integration were fixed 3–5 ms after the beginning of the pulse, once 80% of the Na^+^ current was decayed, and excluding the tail currents. Exocytosis was estimated by the membrane capacitance increment (ΔC) evoked by a depolarizing step (100 ms to +10 mV), according to the Lindau–Neher technique implemented as the “Sine + DC” feature of the PatchMaster software [15]. A sinusoidal wave function (1 kHz, ± 20 mV amplitude) was superimposed on the V_h_. Capacitance increments were acquired by the high time resolution PatchMaster module. To determine ΔC, membrane capacitance was first averaged over 50 ms preceding the depolarization to give a baseline value; this was subtracted to the value estimated after the depolarization, averaged over a 400 ms window, excluding the first 50 ms to avoid contamination by non secretory capacitative transients. Cells in which series resistance changed by more than 20% or holding current exceeded 20 pA were discarded. Membrane potentials were recorded under current-clamp conditions and filtered at 3 kHz. Experiments were performed at room temperature (22–24 °C).

### 4.6. Solutions and Chemicals

Perforated-patch recordings in isolated DRG neurons were performed with an extracellular solution containing (mM) 145 NaCl, 2.8 KCl, 2 CaCl_2_, 1 MgCl_2_, 10 4-(2-Hydroxyethyl)piperazine-1-ethanesulfonic acid, *N*-(2-Hydroxyethyl) piperazine-*N*′-(2-ethanesulfonic acid) (HEPES), and 10 glucose (pH 7.4 adjusted with NaOH; ≈300 mOsm) that was constantly superfused at a rate of approximately 1 mL × min^−1^. Recordings in chromaffin cells were carried out in the Ringer’s saline used to store tissue slices of the adrenal gland (see “Adrenal gland preparation”. Measurements of P2X7 receptor-mediated currents were performed in a Mg^2+^-free extracellular solution. Drugs were added to the superfusion medium or directly applied onto the cell under investigation by pressure ejection from a puffer pipette with an opening of around 3–5 μm placed near (5–10 μm) the cell (adrenal slices), or a multibarrel concentration-clamp device coupled to electronically driven miniature solenoid valves, the common outlet of this was placed within 100 μm of the cell to be patched (isolated DRG neurons). Acetylcholine (ACh), adenosine 5′-triphosphate (ATP), α,β-methylene ATP (α,β-meATP), (2′(3′)-*O*-(4-Benzoylbenzoyl) adenosine 5′-triphosphate (BzATP), P^1^,P^4^-diadenosine tetraphosphate (Ap_4_A), P^1^,P^5^-diadenosine pentaphosphate (Ap_5_A), (3-[[5-(2,3-dichlorophenyl)-1*H*-tetrazol-1-yl] methyl]pyridine hydrochloride (A438079), capsaicin, and capsazepine, were purchased from Sigma-Aldrich, dissolved in distilled water and added to the extracellular solution to reach the concentration indicated. Diinosine pentaphosphate (Ip_5_I; 10 μM), was prepared by the enzymatic degradation of its corresponding diadenosine pentaphosphate (Ap_5_A) using 5′-adenylic deaminase, and purified using reverse-phase chromatography [79]. Agonist drugs were applied for the time indicated by horizontal bars depicted in the figures; antagonists were superfused 2 min before and during application of agonists.

The perforated patch configuration was obtained using amphotericin B (Sigma-Aldrich). Amphotericin B was dissolved in dimethyl sulfoxide and stored at −20 °C in aliquots of 50 mg/mL. The pipette-filling solution used to record voltage-gated Na^+^ and K^+^ currents as well as membrane potentials contained (mM): 145 KCl, 2 MgCl_2_, 0.3 EGTA, 0.3 GTP.Li_3_, 2 ATP.Na_2_, 10 HEPES (pH 7.2 adjusted with KOH; ≈280 mOsm). KCl was substituted for Cs^+^ in experiments aiming to isolate voltage-gated Ca^2+^ currents in an internal solution containing (mM): 90 CsSO4, 55 CsCl, 8 NaCl, 1 MgCl_2_, 15 HEPES (pH 7.2 with CsOH; ≈280 mOsm). Nicotinic, purinergic and capsaicin-activated currents were recorded with the following internal solution (mM): 140 *N*-Methyl-d-glucamine, 5 EGTA, and 10 HEPES (pH 7.2, adjusted with HCl; ≈290 mOsm). Fresh pipette solution was prepared every 2 h. To seal the cells more easily, the patch pipette was immersed for a few seconds into an internal solution without amphotericin B and then back-filled with the internal solution containing amphotericin B (50–100 µg/mL). After sealing, series resistance decreased gradually to reach values below 20 MΩ within 10 min.

### 4.7. Immunohistochemistry

To process for immunolabeling, adrenal glands from Control and CCI animals were removed and fixed by immersion in 4% paraformaldehyde in 0.1 M phosphate buffer (2 h at room temperature). They were then cut using a cryostat (Micron HM 505E, Heidelberg, Germany) into 12 μm-thick sections. Sections were permeabilized for 1 h at room temperature with 0.2% Triton-X100/2% bovine serum albumin (BSA) in phosphate buffer, rinsed with 2% BSA in phosphate buffer and incubated overnight at 4 °C with the following combinations of primary antibodies: (a) rabbit anti-P2X3 (1:100; Alomone, Jerusalem, Israel) and guinea-pig anti-TH (1:500; Synaptic Systems, Goettingen, Germany); (b) rabbit anti-P2X7 (1:100; Alomone) and anti-TH (1:500); (c) goat antibodies against the VAChT (1:50; Santa Cruz Biotechnology, Madrid, Spain) were used either alone or in combination with anti-P2X7 or -P2X3 antibodies; and (d) rabbit anti-TRPV1 (1:100; Abcam, Cambridge, UK) and anti-TH (1:500). Sections were subsequently incubated (1 h at room temperature) with appropriate secondary antibodies conjugated to Alexa 647 (anti-goat, Life Technologies, Madrid, Spain), Alexa 647 (anti-guinea-pig, Sigma-Aldrich) or Alexa 594 (anti-rabbit, Life Technologies) and 561 (anti-rabbit, Jackson Immuno Research Laboratories, West Grove, PA, USA) at 1:500 dilution. Finally, sections were incubated for 10 min in 4′,6-diamidino-2-phenylindole (DAPI), Life Technologies) at a dilution of 1:500, mounted and coverslipped with Fluorsave^TM^ (Merck, Darmstadt, Germany). Primary and secondary antibodies were diluted in phosphate buffer containing 2% BSA and 0.1% Triton-X100. L4, L5 and L6 DRG (only the right ones from CCI animals) were similarly processed for immunohistochemistry. P2X3 or P2X7 receptors and the GFAP were immunoreacted with the above specified anti-P2X3 and anti-P2X7 antibodies as well as a mouse anti-GFAP antibody (1:100; Life Technologies) and a secondary goat anti-mouse antibody conjugated to Alexa 488 (Life Technologies). Stained sections were imaged with a Leica DM IL confocal laser-scanning microscope (Leica Microsystems, Wetzlar, Germany). The specificity of the commercial antibodies has been assessed by absorption tests. Negative controls were carried out by omitting primary antibodies.

### 4.8. Statistics

Data are expressed as the mean ± standard error of the mean (SEM) for the corresponding number of experiments/cells and animals used. Statistics between groups were done using two-way ANOVA for repeated measures, followed by Bonferroni test for post-hoc pairwise comparisons. Paired or unpaired Student’s *t* tests were used to compare means. Differences with *p* < 0.05 were considered significant. GraphPad Prism version 5.0 Software was used (GraphPad Software, San Diego, CA, USA) to perform all statistical analysis.

## Abbreviations

A438079	(3-[[5-(2,3-dichlorophenyl)-1*H*-tetrazol-1-yl]methyl]pyridine hydrochloride
ACh	Acetylcholine
Ap_4_A	P1, P4-diadenosine tetraphosphate
Ap_5_A	P1, P5-diadenosine pentaphosphate
ATP	Adenosine 5′-triphosphate
BSA	Bovine serum albumin
BzATP	(2′(3′)-*O*-(4-Benzoylbenzoyl) adenosine 5′-triphosphate
CCI	Chronic constriction injury
DAPI	4′,6-diamidino-2-phenylindole
DMEM	Dulbecco’s modified Eagle’s Medium-low glucose
DRG	Dorsal root ganglia
GFAP	Glial fibrillary acidic protein
HEPES	4-(2-Hydroxyethyl)piperazine-1-ethanesulfonic acid, *N*-(2-Hydroxyethyl) piperazine-*N*′-(2-ethanesulfonic acid)
i.pl.	intraplantar
Ip_5_I	Diinosine pentaphosphate
min	Minutes
nAChRs	Nicotinic acetylcholine receptors
PWT	Paw withdrawal threshold
Q_Ca_	Integral of the voltage-gated Ca^2+^ current
sEPSCs	Spontaneous excitatory postsynaptic currents
SGC	Satellite glial cells
SNS	Sympathetic Nervous System
TH	Tyrosine Hydroxylase
TRPV1	Transient receptor potential vanilloid-1
VAChT	Vesicular acetylcholine transporter
V_h_	Holding voltage
V_comm_	Command voltage
V_r_	Resting membrane potential
α,β-meATP	α,β-methylene ATP
α9*	α9 subunit-containing
α-RgIA	α-conotoxin RgIA
ΔC	Change in cell capacitance
